# Effects of Inequality on Trust and Reciprocity: An Experiment With Real Effort

**DOI:** 10.3389/fpsyg.2021.745948

**Published:** 2021-12-02

**Authors:** Amalia Rodrigo-González, María Caballer-Tarazona, Aurora García-Gallego

**Affiliations:** ^1^Department of Corporate Finance, Universitat de València, Valencia, Spain; ^2^Department of Applied Economics, Universitat de València, Valencia, Spain; ^3^Laboratorio de Economía Experimental (LEE), Department of Economics, Universitat Jaume I, Castelló de la Plana, Spain; ^4^Instituto Complutense de Análisis Económico, Universidad Complutense de Madrid, Madrid, Spain

**Keywords:** inequality, trust, reciprocity, altruism, real-effort task, experiment

## Abstract

The purpose of this paper is analyzing whether trust and reciprocity are affected by how rich the partner is or how well the partner performed several tasks with real effort. A trust game (TG) experiment is designed with three treatments. First, a baseline Treatment B in which subjects play a finitely repeated TG. Second, in a Treatment H with history, subjects know the partner’s wealth level reached in the past. Third, in a Treatment E with effort the individual endowment with which the TG is played is endogenous and results from the subject’s performance in three different real effort tasks (maths, cognitive and general knowledge related). The data analysis highlights the importance of past wealth levels (Treatment H) as well as endowment heterogeneity (Treatment E), on the actual levels of trust and reciprocity. Specifically, it is observed that the decision of trustors is positively affected by positive past experienced reciprocity. Moreover, trustors are sensitive to how much money the trustee accumulates each round in Treatment H, trusting more the ones that have accumulated less compared to themselves. In contrast with that, it is remarkable in Treatment E that trustors are sensitive to the endowment level of the trustees, trusting more the partners that have got a higher than own endowment, probably considering that a person that performed better in the tasks is a better partner to trust. As far as second players’ behavior, as the amount received from the trustor increases it is less likely that the trustee reciprocates with higher than or with the egalitarian amount. In Treatments H and E, the probability that the trustee reciprocates with higher amount that the one received increases when inequality in endowment/accumulated earnings favors the trustor. Additional results come from analysis of personality archetypes and socio-demographic variables.

## Introduction

The study of human behavior in terms of trust and reciprocity is crucial for understanding the social capital creation that allows achieving goals commonly shared by societies. Experimental and behavioral economics have understood the importance of this issue and have given us a huge spectrum of results in which trust and reciprocity are the focus of the question. Specifically, numerous references analyze the dynamics of trust and reciprocity under different set-ups focusing on the effect of income inequality.

The motivation behind the role played by economic inequality in human behavior is intuitively relevant. The amount of money owned by people is naturally heterogenous, especially because it may have been originated differently and such differences seem to matter a lot. The truth is that human beings care a lot about economic heterogeneity among their peers. In particular, it seems reasonable to hypothesize that humans naturally value more the income created from their own work than the one coming from other non-work-related sources like, for example, inheritance or subsidies. In other words, people care about whether the money comes from own effort or just comes as manna from heaven, and this may affect the willingness to invest and the way of investing the money. And this effect may be stronger in the case that the investment has uncertain returns, especially returns that depend on others’ decisions. This source of economic heterogeneity is considered endogenous.

A different dimension of economic heterogeneity is the one created as a result of being aware of how different my accumulated earnings are along life with respect to my peers. This source of information may be so relevant for economic behavior of people as, for example, to affect the levels of generosity, altruism or even the levels of trust on others in specific environments. In fact, social preferences are relevant in individual decision making, since they are formed by personality factors as well as by social norms. For sure one should differentiate between decisions taken in a situation where all subjects have similar wealth from the situation in which wealth differences exist. Being aware of wealth differences with my peers may wake up fraternity feelings on me and the willingness to equilibrate the imbalance by being active in giving money; or just the opposite may happen, feeling that I deserve more than the others and to make decisions that make our differences even higher. No trivial combinations and results can be found under economic inequalities, and this is the focus of our interest in this research.

The Trust Game (TG) represents a situation that is appropriate to experimentally analyze the effect of facing such economic inequality on subjects’ decisions related to trust. To the best of our knowledge, our work is the first in designing a situation in which trust, reciprocity and altruism are analyzed taking into consideration those sources of economic heterogeneity: the own effort endogenous income inequality and the unequal accumulated earnings. Our design extends the TG experimental literature but tries to cover an empty space in which trust, reciprocity and altruism are analyzed under the influence of heterogeneous initial endowment generated through subjects’ performance in real-effort tasks. The design also considers another source of heterogeneity, the one created by differences in accumulated earnings, which has somehow already been considered in previous literature.

Our purpose is analyzing whether trust and reciprocity are affected by how rich my partner is compared with me or how well the partner performed several tasks with real effort in contrast with my own score. A TG experiment is designed with three treatments. First, a baseline Treatment B in which subjects play a finitely repeated TG (Berg et al., 1995). Second, in a Treatment H with history, subjects know the partner’s wealth level reached in the past. Third, in Treatment E with effort, the individual endowment with which the TG is played is endogenous and results from the subjects’ performance in three different real effort tasks (maths, cognitive, and general knowledge related). Furthermore, our TG version allows for the trustee decision to disentangle reciprocity from altruism, since the decision is double: first, how much of the amount received to return to the trustor and, second, what part of the endowment to give to the trustor.

The data analysis highlights the importance of heterogeneity in earnings levels (Treatment H) as well as in initial endowment (Treatment E) in the last two periods on the actual levels of trust and reciprocity. Specifically, it is observed that the decision of trustors is positively affected by positive past experienced reciprocity. Moreover, trustors are sensitive to how much money the trustee accumulates each round in Treatment H, trusting less the ones that have more compared to themselves. In contrast, it is remarkable the fact that in Treatment E trustors are sensitive to the endowment level of the trustees, trusting more the partners that have got a higher than own endowment, probably considering that a person that performed better in the tasks is a better partner to trust.

As far as the trustee is concerned, his role aims at reducing the wealth gap existing between the two players. Specifically, we take the egalitarian strategy as a reference, meaning that the trustee sends back to the trustor an amount such that his earnings equalize those of the trustor. Three reciprocity levels are taken into consideration: first, second and third levels of reciprocity stand for sending back to the trustor, respectively, a lower, equal and higher amount. In this sense, data reveal that it is more likely that the trustee reciprocates with higher or equal to the egalitarian amount as the trustor decreases the amount sent in the first place. In Treatment H/Treatment E the probability that the trustee reciprocates with higher/equal than/to the egalitarian amount increases when inequality in accumulated earnings/endowment favors the trustor. Previous results provide several tentative explanations to this behavior. For instance, Attanasi et al. (2019) justify the increase in reciprocity in the face of low trust levels as an incentive to raise trust levels in the future. Furthermore, Khalmetski et al. (2015) and Balafoutas and Fornwagner (2017) use a dictator game and found that guilt aversion plays a role in second movers’ decision and, because of that, correlation between transfer and expectations can be negative.

In our design the decision of the trustee is double, so that we can measure not only the reciprocity level, but we also measure the level of altruism when the trustee decide how much of his own endowment to send to the trustor. Results show that the probability of being altruistic for a trustee is independent of the reciprocity decision but it depends positively on the trustor decision as well on his advantage (disadvantage) in endowment (cumulated earnings) with respect to those of the trustor.

The Equality Equivalence Test (EET) has been used in order to classify subjects by personality archetypes. It is worth mentioning that trustors classified as inequality-lovers present significant differences with respect to those classified as altruists. In general, trustees classified as altruists in the EET are trustees that more likely will choose to reciprocate with the egalitarian strategy.

The remainder of the paper is organized as follows. Section “Related Literature” reviews the related literature on trust experiments. Section “Materials and Methods” lists our main research questions and also gives a detailed description of the experimental design. The results are presented in section “Results.” Section “Econometric Analysis” concludes.

## Related Literature

The seminal work by Berg et al. (1995), has been widely used in experimental economics to study trust and reciprocity behaviors. Many authors made some variations on the Berg’s TG in order to stand out other factors involved in cooperative behavior. For instance, it has been found that factors such as experimental protocols and geographical variations or gender, among others, have an effect on trust levels. For example, Johnson and Mislin (2011) find that minor variations in the design protocol (i.e., payment criteria, rate of return or population characteristics) can imply significant changes in share behavior. Their findings suggest that subjects trust less if they are paid randomly and if they play with a simulated counterpart instead of a human. Moreover, trustworthiness decreases when the rate of return is 2 (instead of 3) and when the experiment was run with students. In the same line, in Bornhorst et al. (2010) participants choose their partner to play a TG with some information about each other’s age, gender, nationality and number of siblings. At the beginning of the sessions, authors find differences among participants’ decisions from northern and southern countries in terms of share amounts and type of partner chosen. However, over the course of the game, those cultural differences become blurred. This research evidences that, in spite of the different individual characteristics, trust breeds trust and allows to identify where to find trustworthiness.

One of the aspects which has recently attracted the researchers’ attention is the effect of heterogeneity on trust and reciprocity behaviors. On one hand, the non-experimental literature has long since coincided with the negative effect of individual characteristics heterogeneity on trust levels and cooperative behaviors (Putnam, 2000). On the other hand, recent experimental literature has focused on how wealth heterogeneity implies variations on levels of trust and reciprocity.

Most of the experimental studies agree on the idea that wealth inequality and generalized trust correlate negatively (Gallego, 2016), although aspects such as the availability of information or the direction of inequality regarding the other players should be considered (Andreoni et al., 2017; Xiao and Bicchieri, 2010; Bejarano et al., 2018).

For instance, Lei and Vesely (2010) explore how the inequality in the endowment activates favoritism among the members of the same group, through the TG and the dictator game. They find that this favoritism within the group remains even if there is no longer inequality, concluding that favoritism is activated within members of the same group only in cases in which trustors are classified as rich. However, this favoritism effect decreases but does not disappear when playing under an equitable endowment. Effects of group membership on trust were explored also by Smith (2011a). He found that information about the identity of the other player had positive in-group and negative out-group effects on trust. However, the in-group effect was small and statistically insignificant, while the out-group effect was larger and statistically significant.

The role of an unequal endowment was also explored by Smith (2011b) and Brülhart and Usunier (2012). These authors produce lab-induced players with high and low endowment, and observe which are the behavioral dynamics in the four combinations or profiles of couples. While Brülhart and Usunier (2012) did not find a different behavior when individuals play with rich players or poor players, Smith (2011b) found that subjects with low endowment paired with high endowment subjects showed more trust than subjects in other pairs; in addition, their trust was reciprocated with higher trustworthiness. In the same line, Ciriolo (2007) finds that an unequal distribution of show-up fees may eventually reduce the incentive to cooperate of both players.

Other authors have focused their research on the trustee’s behavior. For example, Xiao and Bicchieri (2010) study inequality aversion when the trustee has lower endowment than the trustor. In this case, it is observed that the trustee’s reciprocity decreases significantly, and the authors associate this effect with inequality aversion. In this line, Rodriguez-Lara (2018) also focus on the trustee’s strategy, but no evidence of inequality aversion is found. They find that in a context of heterogeneous endowment, reciprocity decreases, but not necessarily because of inequality aversion. Conversely, Bejarano et al. (2021a) create an inequality endowment through negative shocks, resulting in a situation where trustors are poorer than trustees. Within this context, the authors observe that inequality increases the levels of both, trust and reciprocity.

Regarding the effect of inequality, information availability seems to be the key point for some authors. For instance, Anderson et al. (2006) conclude that the effect of inequality on trust, in terms of both sign and significance, depends on whether the show-up payments are awarded publicly or privately. In other words, when the induced inequality of payments is awarded privately, the levels of trust decrease; however, when payments are awarded publicly, differences on trust levels are not observed. Inequality has not the same effect on all players, though. Heap et al. (2013) study the inequality effect in a non-market and in a market setting (trust and labor market games, respectively) and found that when it is common knowledge, inequality has a negative effect on trust. In addition, trust in a market setting appears generally more sensitive to the introduction of inequality than in the non-market setting. That is, the wage levels (trust) are on average lower when there is inequality.

Blanco and Dalton (2019) combine a Dictator Game lab experiment with information about the real income stratum of each participant. A positive relation between donations and wealth is shown to be due to the fact that for rich people the experimental endowment has lower real value. They find that the motivation to donate is similar across strata, where the generosity act is explained mainly by warm-glow rather than pure altruism.

Especially inspiring is the work of Greiner et al. (2012) that explore the effect on trust of endogenous as well as exogenous inequality. Authors consider as endogenous inequality the heterogeneity generated along the decisions made in the TG during 20 rounds. The study concludes that with heterogeneous endowment, trust levels remain more stable than in a context of egalitarian endowment. The levels of trust are initially higher in a treatment with equal initial endowments, but these levels of trust decrease over time as the accumulated earnings generated in the game become more heterogeneous. In a treatment with unequal endowments, trust is initially lower than in treatment with equal endowments but the levels remain more stable in comparison with the case of equal endowments.

The design of Fehr et al. (2020) exogenously induces unjust economic inequality after performing a real-effort task, but the payment is not related with the effort nor with the performance in the task. Results show a decline in levels of trust and reciprocity on the extent to which this is deemed fair by participants.

In Bejarano et al. (2018, 2021b), authors analyze the inequality effect on trust and reciprocity both in a context of endowment heterogeneity and inequality generated by random shocks. They find that first-movers send less to second-movers only when the inequality results from a random shock. Moreover, second-movers return less when they are endowed less than the first-mover, regardless of whether the difference in endowments was initially given or occurred after a random shock.

With the exception of Greiner et al. (2012), most of the previous studies referred are devoted to studying the effect of exogenous heterogeneity on trust or reciprocity. In line with Greiner et al. (2012), the present work considers both endogenous and exogenous wealth heterogeneity in the analysis of their effects on trust, reciprocity and altruism levels. Furthermore, our analysis endogenously creates income heterogeneity, generated by a set of real-effort tasks carried out before playing the TG. Higher earnings derived from real-effort task is commonly associated with higher effort, and this has an effect on cooperation decision. As Fehr (2018), Fehr et al. (2020) suggest, the fairness in the income-generating process matters.

Additionally, more recent experimental literature on trust focuses on categorizing individuals based on personal characteristics or motivations. Some of these works have used post-experimental questionnaires with questions to correlate psychological or cognitive characteristics with behaviors observed in the game. This is the case of Corgnet et al.’s (2016) work which combines the decisions in the TG with the results in the Cognitive Reflection Test (CRT). The authors find a positive relationship between cognitive reflection and trusting behavior. In this line, in Bellucci et al. (2019), participants carry out the RSFC test (resting-state functional connectivity) after playing the TG because they were interested in observing the relation between the decisions in TG and results in RSFC. Also, Espín et al. (2016) analyze the relation between the decisions in the TG game and individuals’ social motivation. Their research applies both the Dictator Game and a dual-role Ultimatum Game to identify individuals’ social preferences for altruism, spitefulness, egalitarianism, and efficiency. They find considerable heterogeneity in the TG decisions’ motivation. Furthermore, in Attanasi et al. (2013, 2019) authors use pre and post-experimental questionnaires to correlate players’ characteristics with their decisions in the TG. The purpose of introducing the questionnaires was to classify trustees as guilt averse or selfish. Trust increases with guilt sensitivity and the reputation effect is very strong.

Other authors who want to investigate motivations of trust, try to isolate the behavior that really indicates a trust decision. A good example of this is Chetty et al. (2020). They focus on identifying risk-trust relationships by using a risk-preference task. They conclude that attitudes to risk may partly confound the measurement of trust. In this line, Cox et al. (2016) uses different treatments of the investment game to categorize individuals according to their social preference, and then, analyzing their decisions on the TG, in order to isolate effects as vulnerability or inequality aversion from trust.

With this background in mind, the present paper analyses the effect of income inequality on trust and reciprocity. The income heterogeneity comes from two different sources. On one hand, it is endogenously generated through the TG-repeated decisions. On the other, the inequality comes from a heterogeneous endowment generated from real-effort tasks performed before playing the TG. Different experimental treatments are designed in order to isolate these effects. Finally, inspired by the work of Cox (2004) and Anderson et al. (2006), our design allows to disentangle the second-player decision in the TG between reciprocity and altruism.

## Materials and Methods

This section describes in detail the experimental design and also motivates the research questions.

### Experimental Design

The experiment is divided in three treatments, all having in common that participants play the Trust Game. Therefore, before describing the details of each treatment, the version of the Trust Game implemented in our treatments is exposed.

#### Our Trust Game

Following the version of Berg et al. (1995), a trustor (sender) and a trustee (receiver) are endowed with the same amount of money *E*. The trustor decides which part (in absolute value) *x* ϵ (0, *E*) of the endowment to send to an anonymous trustee. The amount *x* is then multiplied by *n* = 3 in the trustee’s hands. After the trustor’s decision is observed, the trustee decides about two (absolute) amounts to return to the trustor^1^ :

1. Amount *y*_1_ ϵ (0, 3*x*) to return to the trustor.
2. Amount *y*_2_ ϵ (0, E) to send to the trustee.

**FIGURE 1:**
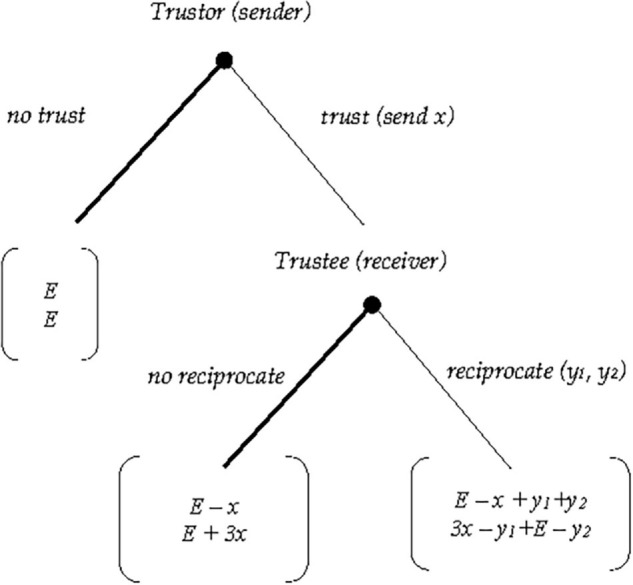
Extensive form of the one-shot TG with fixed endowment.

Consequently, the final payoff for the trustor is π_*or*_ = *E*−*x* + *y_1_* + *y*_2_, and that of the trustee equals π_*ee*_ = 3*x*−*y_1_* + *E* −*y*_2_. Figure 1 shows the extensive form of the TG version just described.

This game has a unique subgame perfect Nash equilibrium in (“*no trust,” “no reciprocate”*) and therefore, neither trust nor reciprocity is a possible result under the assumptions of rationality and selfishness of both players.

Our subjects played this TG repeatedly during 12 rounds. Each round each subject was randomly matched with a different participant in the same session. Each session had 5 groups of 8 people each, so that each group can be considered an independent observation in our analysis.

#### Treatments

The three treatments of our experiment are the following (see Table 1):

**TABLE 1:** Experimental treatments.

Treatment	Endowment	Sessions	Subjects	Females
Baseline -Treatment B	50 ExCUs	2	80	47.50%
History -Treatment H	50 ExCUs	2	80	41.25%
Effort -Treatment E	[10, 100] ExCUs	2	80	48.75%

*Treatment B. Baseline* treatment in which subjects play the TG during 12 periods with fixed initial endowment, random matching and fixed roles. At the end of each round, each player receives feedback on own payoffs and accumulated payoffs in that specific round. No feedback about the partner’s earnings is given at all.
*Treatment H*–Treatment with *History.* It is the same as the baseline with subjects receiving at the end of each period, feedback about own as well as the partner’s total earnings accumulated in the past.
*Treatment E*–Treatment with *Effort*. This treatment differs from the other treatments in that the initial endowment is endogenous. In this treatment, subjects play first an Effort Task (with three sub-tasks). The endowment of each subject depends on the performance of the subject in the three tasks. In particular, we established a linear relation between the endowment and the final score so that a certain level of heterogeneity was assured.

#### Experimental Session

Two sessions were run of each treatment. Each experimental session included different stages, most of them common to all treatments. Table 2 shows in detail the stages of a session:

**TABLE 2:** Structure of an experimental session.

Stage	Decision making
0	Real-effort tasks (only in Treatment E)
1st	EET-pre
2nd	Trust Game
3th	EET-post
4th	Socio-demographic questionnaire
5th	Questionnaire on trust and reciprocity

*Stage 0. Real-effort tasks (only for Treatment E)*

Subjects performed three individual tasks. The first is related with visual search and consisted in counting ones; the second was of cognitive nature and subjects had to sum 3-digit numbers; the third was miscellaneous and consisted in answering multiple choice questions on general knowledge. In the following we describe each task in more detail:

*– Task 1. Counting number of ones* (Mohnen et al., 2008; Abeler et al., 2011): In a sequential way, each computer screen showed to the subject a 6×6 matrix with randomly ordered 0 and 1s. The subject had to count and write the number of 1s, with no feedback about whether the answer was correct. The participants solved as many matrices as possible during 3 min.
*– Task 2. Summing 3-digit numbers* (Niederle and Vesterlund, 2007): In a sequential way, subjects had to add four 3-digit numbers, without getting any feedback about whether the answer was correct. The participants summed as many series as possible within a time period of 3 min.
*– Task 3. General Knowledge* miscellaneous quiz questions about society, history, geography, maths etc., general knowledge that everyone may acquire through formal education (Coane and Umanath, 2021). The task was programmed with a maximum of 50 questions. Each subject had to answer as many questions as possible during 2.5 min.^2^

*Stages 1 and 3. The Equality Equivalence test (EET-pre and EET-post)*

The EET (also known as EE-test) was developed by Kerschbamer (2015) to elicit a subject’s distributional preference type. It is based on two panels with 5 binary choices that affect both own payoff and other’s payoff (see Table 3). In the first panel (benevolence behind), decisions are made between receiving the same payoff as the other or a lower one (disadvantageous inequality, x-list). In the second panel (benevolence ahead), decisions are made between receiving the same or a higher payoff as the other (advantageous inequality, y-list). The structure of the test is such that, in order to fulfill the m-monotonicity property, a rational subject decides to switch her decision from equality to inequality once at most.

**TABLE 3:** Equality Equivalence Test (EET).

LEFT				RIGHT	
You receive	Another person receives			You receive	Another person receives
**Benevolence behind**					
3.2	5.2	LEFT	RIGHT	4	4
3.6	5.2	LEFT	RIGHT	4	4
4	5.2	LEFT	RIGHT	4	4
4.4	5.2	LEFT	RIGHT	4	4
4.8	5.2	LEFT	RIGHT	4	4
**Benevolence ahead**					
3.2	2.8	LEFT	RIGHT	4	4
3.6	2.8	LEFT	RIGHT	4	4
4	2.8	LEFT	RIGHT	4	4
4.4	2.8	LEFT	RIGHT	4	4
4.8	2.8	LEFT	RIGHT	4	4

This test reveals how benevolent the subject is in the domains of disadvantageous and advantageous inequality. We use this test in order to control for social archetypes. Computing the (*x-score*, *y-score*) as described in Holzmeister and Kerschbamer (2019, p. 219), we are able to identify four behavioral archetypes^3^ : altruist (b, b), spiteful (m, m), inequality loving (b, m), and inequality adverse (m, b).

In all treatments, this test was performed by the subjects before (EET-pre) and after (EET-post) playing the TG. Our motivation is that the decisions made in the TG may affect the distributional preference of the subjects. In Treatment E we informed the subjects that this task had no relation whatsoever with part “zero” of the session–the real-effort tasks. In each of the two performances of this test, each subject is randomly matched with another anonymous participant in the room.

*Stages 4 and 5. Questionnaire*

In the final part of the session, subjects had to answer a questionnaire that was divided in two parts.^4^ In the first part, questions related to socio-demographic issues like gender, age, studies, job, and housing were formulated. In the second part, the questions focused on personality traits related to trust and trustworthiness (Evans and Revelle, 2008),^5^ negative and positive reciprocity (Caliendo et al., 2012, 2014),^6^ and empathy (Spreng et al., 2009).^7^

#### Participants

The sessions were run within the time period November 2018 - November 2019 in the Laboratorio de Economía Experimental (LEE), at the Universitat Jaume I in Castellón (Spain). The experiment was programmed in z-Tree (Fischbacher, 2007). Participants were all students from several degrees (engineering, health science, humanities, social sciences, etc.) taught at that University and were recruited using ORSEE (Greiner, 2015). Two sessions of 40 subjects per treatment were run, with a total of 240 participants (80 per treatment). Each session lasted around 90 min and average payoffs were 14 euros per subject.

### Research Questions

The central objective of our study is to analyze the effect of economic inequality in human decisions related to trust, reciprocity and altruism. The results by Berg et al. (1995) constitute our reference’s point in the formulation of our research questions. Throughout the paper the absolute amount of money the trustor sends to the trustee is denoted as “trust level”, and the absolute amount the trustee sends back to the trustor from the money received (initial endowment) is denoted as “reciprocity level” (“altruism level”). Four are the main research questions of our design:

**RQ1.** The decisions of the trustor in the TG are expected to show that the level of trust observed in one period depends on the reciprocity experienced in the last round, and this relation has a positive sign.

This is a result expected in any TG, independently of the treatment. In fact, it is assumed that one of the motivations of the trustor for sending a positive amount to the trustee is her expectations about receiving some amount back from him.^8^

**RQ2.** Compared with the baseline, in the treatment with heterogeneous endowment (Treatment E) the trustor sends, on average, lower amounts to the trustee. However, no general effect is expected on the trustee’s behavior.

This research question motivates our Treatment E. In fact, deciding on how much money to send to an anonymous partner may be affected by the origin of the initial endowment. Specifically, if the endowment comes from performing several tasks, this fact is expected to play a significant role in trustor’s decisions in comparison with the situation in which the endowment comes as manna from heaven. However, this endowment heterogeneity is not expected to play a role in trustees’ behavior, since the reciprocity level is considered to be purely affected by the decision of the corresponding trustor.

**RQ3.** In Treatment E where subjects play with unequal initial endowment, to have higher endowment positively affects trust and reciprocity levels.

Playing Treatment E results in “endogenous inequality,” since the endowment depends on the performance of the subject in three real-effort tasks. We speculate that the origin of the inequality may have an effect on trustors and, specifically, we believe that having a higher endowment makes the trustor/trustee more likely to send a higher amount to the trustee/trustor.

**RQ4.** In Treatment H, to be the one with higher cumulated earnings positively affects trust and reciprocity levels.

Treatments H and E may result in economic inequalities among the subjects. Specifically, playing Treatment H results in economic inequality given that -except for the first period- subjects may end up with different cumulated earnings. The same argument described for RQ3 holds here in the sense that, for a trustor/trustee, being the one with higher accumulated earnings makes it more likely to send a higher amount to the trustee/trustor. Individual experiences, characteristics and situations can influence trust levels. Then, a situation of economic advantage/disadvantage can condition trust and reciprocity decisions. Alesina and La Ferrara (2002), in a broad analysis of individual and community characteristics that influence how much people trust each other, point to the economic unsuccessfulness in terms of income as one of the main factors that reduce trust levels. Similarly, we can expect that individuals with more resources tend to trust and reciprocate more (Yan and Miao, 2007). Thus, both in RQ3 and RQ4 we expect a positive effect of economic advantage on trust and reciprocity.

## Results

In this section we present the results of our data analysis. First, we summarize the non-parametric analysis, carrying out a general perspective of the data obtained and offer some preliminary insights. After that, we show the adjustment of an econometric model for the behavior of both types of players, trustor and trustee, in which some related variables identified during the experiment are included.

### Real-Effort Tasks (Only in Treatment E)

The score in a task is the sum of total correct answers. The global score in each task is computed by the sum of the three scores weighted by the value of a correct answer. Because the difficulty level is heterogenous^9^ among tasks, task 1 is taken as the reference task. The tasks requiring higher effort were given a higher weight. Weights of 25, 40, and 35% were applied for task 1, task 2 and task 3, respectively. Thus, a correct answer is task 1 has a value of 1 point, of 1.6 points in task 2, and of 1.4 in task 3. Mistakes were allowed in the three tasks but incorrect answers were not considered for the final score. The total score for each subject was therefore calculated as:


$$\mathrm{Score}=\left(1\times N_{1}\right)+\left(1.6\times N_{2}\right)+\left(1.4\times N_{3}\right)$$

Where N_*i*_ is the number of correct answers in task i.

On average, making a ranking in the negative domain, task 3-general knowledge questions was performed the worst, with an average error rate of 32.26%. Task 2-summing four 3-digits numbers was the second in the ranking, reaching 30.53% of incorrect answers. Task 1-counting ones was, as expected, the best performed, with 11.86% rate of average error (see Table 4).

**TABLE 4:** Average error rates, global score, and final endowment in the real-effort tasks, by gender.

	Task 1 25%	Task 2 40%	Task 3 35%	Global Score	Endowment in ExCUs	Obs.
Females	10.37% (0.12)	30.43% (0.22)	35.56% (0.15)	29.17 (9.81)	51.51 (12.24)	39
Males	13.28% (0.14)	30.63% (0.23)	29.31% (0.14)	35.69 (10.64)	61.67 (17.51)	41
All	11.86% (0.13)	30.53% (0.22)	32.26% (0.15)	32.51 (11.25)	56.72 (16.74)	80

*Std. dev. in parenthesis. Rates of standard deviation error are expressed in decimal numbers.*

The system then had to calculate the initial endowment of each subject in order to start the part dedicated to playing the TG. The endowment of each participant after performing the tasks was calculated in such a way that differences in performance could guarantee enough heterogeneity among endowments in the total population. More specifically, all endowments were within a closed interval in which 10 ExCUs was the minimum value and 100 ExCUs the maximum. Specifically, the endowment of each subject was calculated as E_i_ = [10 + (100–10) score/max score].

### Final Questionnaire’s Results

The second part of the final questionnaire consists in answering questions about personality traits related to trust, trustworthiness, negative and positive reciprocity, and empathy. Table 5 reports some statistics about this data analysis. Specifically, an equal weighted index is computed on the 4-point Likert items of questions corresponding to each category. The categories are: trust, trustworthiness, reciprocity and empathy. Looking for any gender effect, significant differences are found only in negative reciprocity and empathy categories. According to the rank-sum M-W test, males (females) show a higher (lower) score than females (males) with a probability of 0.620 (0.626) in negative reciprocity (empathy).

**TABLE 5:** Summary of personality traits, by gender.

Index (%)	Interpersonal trust	Intrapersonal trustworthiness	Positive reciprocity	Negative reciprocity	Empathy	Obs.
Females	2.74 (0.36)	3.15 (0.31)	3.46 (0.49)	1.81 (0.57)	3.25 (0.30)	110
Males	2.74 (0.33)	3.11 (0.38)	3.49 (0.49)	2.07 (0.64)	3.10 (0.32)	130
All	2.74 (0.35)	3.13 (0.35)	3.48 (0.49)	1.95 (0.63)	3.17 (0.32)	240

**Ranksum M-W test**	z = 0.454 *p* = 0.6500	z = −0.664 *p* = 0.5067	z = 0.520 *p* = 0.6032	z = 3.276 *p* = 0.0011	z = −3.382 *p* = 0.0007	
Males have a higher score than Females with probability	0.475	0.517	0.519	0.620	0.374	

**Sign test**	Low Median < 3	High Median > 3	High Median > 3	Low Median < 3	High Median > 3	
Females	*p* = 0.0000	*p* = 0.0000	*p* = 0.0000	*p* = 0.0000	*p* = 0.0000	
Males	*p* = 0.0000	*p* = 0.0008	*p* = 0.0000	*p* = 0.0000	*p* = 0.0060	

*Average values and standard deviation in parenthesis. Index computed as an average of items in each category.*

### Equality Equivalence Test Performance

**FIGURE 2:**
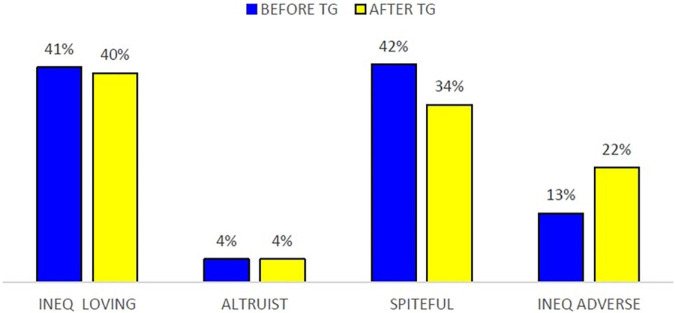
Frequency of EET archetypes before and after playing the TG.

**FIGURE 3:**
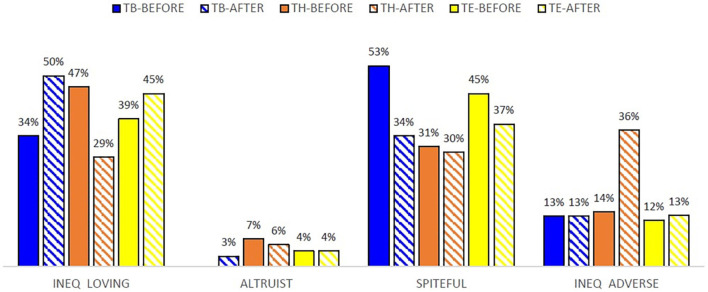
Frequency of EET archetypes before and after the TG, per treatment.

This test allows us to identify four archetypes in our data sample: “inequality loving,” “spiteful,” “inequality adverse,” and “altruist.” A possible TG-effect^10^ on participants’ individual choices in the EET is analyzed. Such effect is represented as a change in the percentage of participants assigned to each archetype according to their individual choices. Figure 2 shows the percentage of each archetype before and after playing the TG. It is observed that the archetypes “spiteful” and “inequality adverse” experience a significant change after playing the TG: a higher number of “inequality adverse” participants are found after playing the TG, whereas the “spiteful” type notably decreases. This can be observed in Figure 3, showing the presence of archetypes in each treatment before and after playing the TG.

Differentiating by treatment (Figure 3), in Treatment B it is detected a general TG-effect on individual choices in EET with major contributions to symmetric payoffs by “inequality loving” and “spiteful” archetypes. It is recorded an increase of 15.79 percentage points in inequality loving participants, and this fact is clearly explained by the decrease in spiteful participants. Observe that in Treatment H the “inequality loving” and “inequality averse” the archetypes contributing to the TG effect most. Specifically, the participants classified as “inequality loving” fall 18.5 percentage points after playing the TG, and the ones classified as “spiteful” fall 1.4 percentage points. In contrast, the “inequality adverse” increase 21.40 percent points. In Treatment E, the TG effect on individual choices is negligible. In fact, the increase of 6.6 percentage points in inequality loving is offset by the decrease of 8 percentage points in spiteful participants.

The above evidence allows confirming that providing participants with information about the partner’s cumulated earnings during the finite repeated TG has a significant effect on the EET choices. In fact, Treatment H shows this effect especially intense on the players who were labeled as “inequality lovers” in the pre-TG then converted in “inequality adverse” in the post-TG. Our interpretation is that participants’ social preferences reflected on the EET are sensitive to the -maybe negative- experience playing the TG. Interestingly, no TG-effect is found on EET when participants earn their initial endowment with their own effort in Treatment E.

### Trust, Reciprocity and Altruism: A Non-parametric Analysis

This subsection presents the results from a non-parametric analysis implemented on trust, reciprocity and altruism decisions.

#### Trust

**FIGURE 4:**
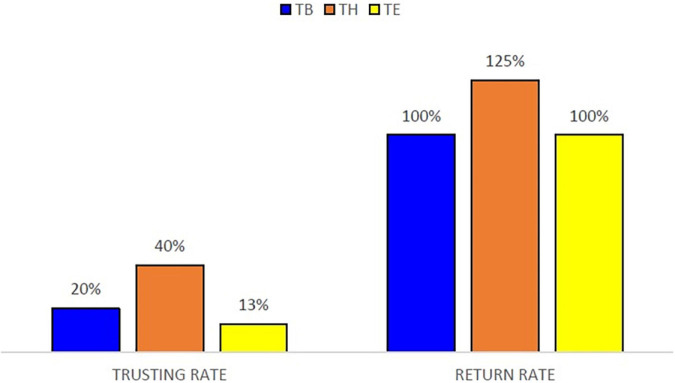
Median decisions in the TG, per treatment.

In order to make the three treatments comparable, the decision of trust is measured as the percentage of the initial endowment, what we call *trusting rate* (see Figure 4).

A first general result is that significant differences among treatments are found with respect to trust, implying that the decisions of the trustor are endowment as well as cumulated earnings dependent. Observe in Figure 4 that in median values, the trustor sends 20% of the endowment in Treatment B, 40% in Treatment H, and 13% in Treatment E. That is, in comparison with the baseline, trust is significantly lower when the initial endowment is endogenously determined through real-effort tasks, but significantly higher in the case the trustor knows the cumulated earnings of the corresponding trustee before deciding in the next period (see Table 6).

**TABLE 6:** Testing treatment and gender effects on TG decisions.

	Treatment differences			Gender differences (males vs. females)		
Trusting rate	z_BH_ = −3.820 *p* = 0.0001	z_BE_ = 4.743 *p* = 0.0000	z_HE_ = 8.512 *p* = 0.0000	z_B_ = 6.109 *p* = 0.0000	z_H_ = 2.663 *p* = 0.0078	z_E_ = −0.477 *p* = 0.6332
Return rate	z_BH_ = −2.267 *p* = 0.0234	z_BE_ = −0.157 *p* = 0.8750	z_HE_ = 1.985 *p* = 0.0471	z_B_ = 0.801 *p* = 0.4234	z_H_ = −1.846 *p* = 0.0648	z_E_ = 2.311 *p* = 0.0208
Reciprocity	z_BH_ = −3.622 *p* = 0.0003	z_BE_ = −0.780 *p* = 0.4354	z_HE_ = 2.745 *p* = 0.0060	z_B_ = 0.165 *p* = 0.8692	z_H_ = −0.330 *p* = 0.7417	z_E_ = 1.163 *p* = 0.2447
Altruism	z_BH_ = −0.207 *p* = 0.8360	z_BE_ = 3.619 *p* = 0.0001	z_HE_ = 3.207 *p* = 0.0013	z_B_ = 0.440 *p* = 0.6602	z_H_ = −3.499 *p* = 0.0005	z_E_ = −1.051 *p* = 0.2932

*The test applied is the Wilcoxon rank-sum test for two independent samples.*

Focusing on Treatment H, trustors with higher cumulated earnings than their partners, send a significantly higher (in median) percentage of their endowment to the trustee compared to trustors with equal or lower cumulating earnings than their partners. The opposite result is obtained when extrapolating to Treatment E, i.e., the trustor rate (in median) is lower for trustors with higher endowment than their (see Table 7).

**TABLE 7:** Testing the effect of earnings/endowment inequality on TG decisions.

	Treatment H	Treatment E
Trusting rate	WC signed-rank test: z = −3.113, *p* = 0.0019 Left-sided sign test: *p* = 0.0003	WC signed-rank test: z = 2.534, *p* = 0.0113 Right-sided sign test: *p* = 0.0171
Return rate	WC signed-rank test: z = 1.023, *p* = 0.3065 Two-sided sign test: *p* = 0.6089.	WC signed-rank test: z = −0.521, *p* = 0.6022 Two-sided sign test: *p* = 0.3997
Recipro-city	WC signed-rank test: z = 0.691, *p* = 0.4898 Two-sided sign test: *p* = 0.8974	WC signed-rank test: z = −2.376, *p* = 0.0175 Left-sided sign test: *p* = 0.0059
Altruism	WC signed-rank test: z = 2.115, *p* = 0.0344 Right-sided sign test: *p* = 0.0135	WC signed-rank test: z = 1.595, *p* = 0.1106 Two-sided sign test: *p* = 0.3560

*The tests are Wilcoxon signed-rank test for two dependent samples and sign test. The difference between two groups of subjects is tested: a group with those with no advantage with respect to the partner, and another group with advantage. The groups change in each period, and therefore samples are not independent.*

The same Table 6 shows the comparison among treatments of the gender of the trustor. Observe that in Treatment B and Treatment H females send, in median, significantly lower amounts than males.^11^ This is very much in line with several previous results in the literature on trust (Buchan et al., 2008; Dittrich, 2015).

#### Reciprocity and Altruism

It has been already mentioned in section “Materials and Methods” that our design includes two decisions for the trustee: a reciprocity decision that accounts for the amount sent back to the trustor from the total amount received; as well as an altruism decision that accounts for the amount sent to the trustor from the initial endowment. The analysis of reciprocity and altruism are measured using the *return rate*, defined as the total amount sent by the trustee divided by the amount sent by the trustor.

Figure 4 shows the return rate per treatment. In median values, the return rate is 100% in Treatment B and Treatment E, indicating that the trustor sends and receives the same amount. Furthermore, in the treatments with heterogeneity, Treatments H and E, the return rate is not significantly different independently on the advantage/disadvantage that it may exist in cumulated earnings (Treatment H) or endowment (Treatment E) with respect to the partner (see Table 7).

**FIGURE 5:**
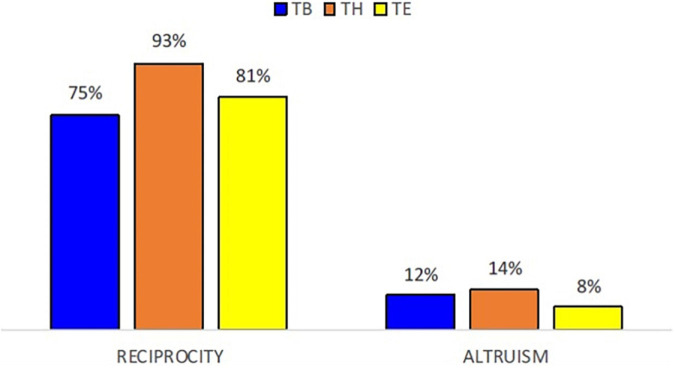
Average levels of reciprocity and altruism in the TG, per treatment.

Figure 5 presents the trustees’ reciprocity and altruism decision separately, in average percentage. It is found that the reciprocity decision is higher in Treatment H than in the other two treatments, on average as well as in median values. Furthermore, Treatments B and E do not show significant differences concerning reciprocity. This may indicate that reciprocity is not primarily determined by inequality on initial endowment, but by inequality built as the game is played and players are aware of the information about the other’s cumulated earnings. It seems therefore that the reciprocity decision is cumulated earnings-dependent.

Regarding altruism decisions, treatment significant differences are found only between Treatment E and the other two treatments, indicating that inequality in the initial endowment is relevant as far as the altruism decision in the TG is concerned (see Table 6). Looking specifically at each treatment, in Treatment E it is observed that the reciprocity decision is significantly higher when trustees have superior endowment than their partners compared to trustees with equal or inferior endowment. On the contrary, in Treatment H the altruism decision of trustees with equal or lower cumulated earnings than their partner is significantly higher than that of trustees with higher cumulated earnings compared to their partner (see Table 7).

Contrary to the role of the trustor, the significant gender differences with respect to the role of the trustee are found in Treatment E. Females send back, in median, significantly lower amounts than males. In Treatment H the same effect is found but the significance is weak. A between treatments analysis shows that females are found to be significantly more altruistic in Treatment H than in the other two treatments.

## Econometric Analysis

In this section we use multivariate regression models to enrich the previous non-parametrical analysis with additional interesting results non-captured by a non-parametric analysis.^12^ Table 8 contains the definitions of both dependent and independent variables. Specifically, the trustor’s decision is modeled by using a multivariate linear regression model; the trustee’s double decision is estimated through two probability models. On one hand, the reciprocity decision is modeled by a 3-level ordered logit model that estimates the probability of each reciprocity level. On the other hand, the altruism decision is modeled by a binary logit model. All models are included in Table 9. Observe in the table that the dependent variables are “trusting rate” (column 1), “reciprocity level” (columns 2–5), and “altruism level” (columns 6–7). For probability models, marginal effects are also shown (columns 3–5, and 7). Concerning ETT data, the results in the table have been calculated under the hypothesis that the m-monotonicity property in the decisions of the EET-pre, is fulfilled.^13^

**TABLE 8:** Definition of variables.

Dependent variables
*Trusting rate*: Amount sent by the trustor/Endowment (*x*/E)
3-level reciprocity variable: {L1, L2, L3} {L1, L2, L3}
– L1: Reciprocity amount (*y*_1_) is smaller than the egalitarian amount
– L2: Reciprocity amount (*y*_1_) equals the egalitarian amount
– L3: Reciprocity amount (*y*_1_) is higher than the egalitarian amount
*Egalitarian amount:*
– In Treatment B: *y*_1_ = 2*x*
– In Treatment H: *Max* {(Gm–Go)/2 + 2*x*, 0} ≤ 3*x*; Gm denotes own (m stands for myself) cumulated earnings and Go is the other’s cumulated earnings
– In Treatment E: *Max* {(Em–Eo)/2 + 2*x*, 0} ≤ 3*x*; Em denotes own (m stands for myself) initial endowment and Eo is the other’s endowment
*Altruism*: Binary variable (taking value 0 if the amount sent from the endowment (y_2_) = 0; or 1 if the amount sent is (y_2_) > 0)

**Independent variables**
*Trustor amount* (x): amount sent by the trustor
*Reciprocity amount* (y_1_): amount returned by the trustee from the total amount (3x) received from the trustor a Altru
Total returned amount lag = 1: total amount sent by the trustee in period t-1
Total returned amount lag = 2: total amount sent by the trustee in period t-2
*Economic inequality*:
– Max{Em – Eo, 0}: Own (m stands for myself) initial endowment (Em) is higher than the other’s (Eo)
– Max{Eo – Em, 0}: The other’s initial endowment (Eo) is higher than mine (Em)
– Max{Gm – Go, 0}: Own (m stands for myself) cumulated earnings (Gm) are higher than the other’s (Go)
– Max{Go – Gm, 0}: The other’s cumulated earnings (Go) are higher than mine (Gm)
*Gender*: dummy variable (0-Male, 1-Female)
*EET-types*: dummy variable (Spiteful, Inequality-lovers, Inequality-averse, Altruist)
*Treatments*: dummy variable (Treatments B, H and E)
*Personality related questions*: 4-point Likert scale (1 strongly disagree, 2 disagree, 3 agree, 4 strongly agree)
– I always act fairly with others. (Trustworthiness)
– If you deal with strangers it is better to be careful before trusting them. (Trust)
– I go out of my way to help someone who was previously nice to me. (Reciprocity)
– I think most people lie to take advantage of others. (Negative Trust)
– I would never evade my taxes. (Trustworthiness)
– If someone offends me, I will offend them. (Negative Reciprocity)

**TABLE 9:** Econometric models for trustors (1) and trustees (2–7).

	1	2	3	4	5	6	7
	Trusting rate	Recipr. Level	L1	L2	L3	Altruism	
	COEF	COEF	ME	ME	ME	COEF	ME
Trustor amount (x)		−0.2446***	0.0237***	−0.0156***	−0.0081***	0.0495***	0.0054***
		(0.0712)	(0.0050)	(0.0043)	(0.0011)	(0.0171)	(0.0019)
Reciprocity amount (y)						–0.0141	–0.0016
						(0.0133)	(0.0015)
Total returned amount lag = 1	0.0041***						
	(0.0007)						
Total returned amount lag = 2	0.0033***						
	(0.0004)						
Max{Em – Eo, 0}	−0.0009***	−0.0535***	0.0053***	−0.0034***	−0.0018***	0.0301***	0.0033***
	(0.0002)	(0.0112)	(0.0012)	(0.0007)	(0.0006)	(0.0044)	(0.0005)
Max{Eo – Em, 0}	0.0012***	0.0897***	−0.0087***	0.0057***	0.0030***	–0.0113	–0.0012
	(0.0001)	(0.0230)	(0.0018)	(0.0015)	(0.0005)	(0.0129)	(0.0014)
Max{Gm – Go, 0}	–0.0002	0.0014*	−0.0001*	0.0001*	0.00005*	−0.0021***	−0.0002***
	(0.0002)	(0.0007)	(0.0001)	(0.0000)	(0.0000)	(0.0008)	(0.0001)
Max{Go – Gm, 0}	−0.0003***	0.2415***	−0.0234***	0.0154***	0.0080***	0.0363	0.0040
	(0.0001)	(0.0568)	(0.0038)	(0.0034)	(0.0010)	(0.0258)	(0.0029)
Female	–0.0290	–0.0579	0.0056	–0.0037	–0.0019	0.5126	0.0566
	(0.0333)	(0.1564)	(0.0153)	(0.0100)	(0.0053)	(0.7047)	(0.0765)
Inequality loving	0.0850***	0.0963	–0.0095	0.0063	0.0032	–0.5867	–0.0647
	(0.0298)	(0.1392)	(0.0137)	(0.0090)	(0.0048)	(0.6199)	(0.0701)
Inequality averse	0.0073	–0.5331	0.0488***	−0.0340***	−0.0149***	0.0965	0.0107
	(0.0374)	(0.1273)	(0.0124)	(0.0084)	(0.0049)	(0.7786)	(0.0863)
Altruist	0.0563	1.3755**	−0.1430**	0.0783***	0.0647	–0.6933	–0.0763
	(0.0542)	(0.6915)	(0.0664)	(0.0283)	(0.0398)	(0.2079)	(0.2229)
Treatment H	0.0505	0.0260	–0.0024	0.0017	0.0008	0.3650	0.0401
	(0.0455)	(0.3729	(0.0348)	(0.0239)	(0.0109)	(0.8688)	(0.0939)
Treatment E	−0.1084***	0.4831	–0.0469	0.0307	0.0162	0.1939	0.0212
	(0.0401)	(0.3967)	(0.0386)	(0.0248)	(0.0141)	(0.7660)	(0.0833)
I always act fairly with others. (Trustworthiness)	0.0775***						
	(0.0203)						
If you deal with strangers it is better to be careful before trusting them. (Trust)	–0.0181						
	(0.0220)						
I go out of my way to help someone who was previously nice to me. (Reciprocity)		0.2122	–0.0206	0.01353	0.0070*		
		(0.1419)	(0.0132)	(0.0091)	(0.0042)		
I think most people lie to take advantage of others. (Neg.Trust)						−0.9156***	−0.1008***
						(0.3336)	(0.0393)
I would never evade my taxes. (Trustworthiness)						0.2083	0.0229
						(0.4205)	(0.0456)
If someone offends me, I will offend them. (Neg. Reciprocity)						−0.9748***	−0.1073***
						(0.2299)	(0.0233)
Constant	0.0164					2.3411	
	(0.0852)					(2519)	
Cutoff point for L1		0.8391*					
		(0.5002)					
Cutoff point for L2		3.5255***					
		(0.6919)					
σ_u_^2^ (panel-level variance)		0.0576					
		(0.0958)					
σ_u_ (panel-level deviation)	0.0783					2.8251	
						(0.3270)	
σ_e_ (error term deviation)	0.1673						
ρ = σ_u_^2^/σ_u_^2^ + σ_e_^2^	0.1796					0.7081	
						(0.0478)	
R^2^ (overall)	0.6359						
Log pseudolikelihood		–465.92				–495.98	
Wald χ^2^	687.61***	7244.36***				4331.13***	
Number of observations	920	1152				1152	
Groups	92	96				96	

All regressions are estimated with random-effects and cluster–robust standard errors for panels nested within groups. The trust model is estimated as a linear regression with GLS estimation. COEF indicates regression coefficient, and ME indicates marginal effect. Standard errors in parentheses. Independent variables are defined in Table 8. *, **, *** indicate statistical significance at the 10, 5, and 1% levels, respectively.

### Trustors’ Behavior

Table 9 shows our estimation for the trustors’ behavior: a lineal model by GLS with random-effects and cluster–robust standard errors for panels nested within groups.

We find a positive and significant relationship between the trustor’s decision in the current period *t* and the trustee’s decision not only in the previous period (*t-1*), but also the previous to the previous period (*t-2*). Our first result summarizes this general finding:

***Result 1*** Independently on the treatment, the trustor’s decision each round is influenced by her recent interaction with the correspondent trustee. In each specific round, the higher the amount returned by the trustee in the previous (up to two) period(s), the higher is the trust transmitted by the trustor, thus sending a higher amount.

However, looking further in the treatments with inequality (Treatments H and E) we search the possible that the difference between own and the partner’s initial endowment may have on the trustor’s decision, the evidence splits up. First, with respect to TE, when the trustor’s endowment is greater than the trustee’s (Em – Eo > 0), the corresponding regression coefficient presents a significant negative sign (−0.09%, *p* = 0.001), which is aligned with the non-parametric evidence commented in the previous subsection. However, in the opposite case (Eo – Em > 0), the coefficient is significant and positive (0.12%, *p* = 0.000). Therefore, our second result states that:

***Result 2*** When the trustor’s endowment is higher (lower) than the trustee’s, the trust level is negatively (positively) affected, sending less (more) money to the partner.

Thus, our RQ3 is partially confirmed, since it holds only for the case in with the trustor has lower endowment than the trustee.

Second, looking at Treatment H, also mixed are the results obtained when looking at the effect of the differences between own and the other’s accumulated earnings. In particular, observe in Table 9 (column 1) the corresponding regression coefficient is negative and no significant (−0.02%, *p* = 0.442) when the cumulated earnings of the trustor are higher than those of the trustee (Gm-Go > 0). Moreover, when the contrary happens, i.e., Go – Gm > 0, the coefficient is negative (−0.03%) and statistically significant (*p* = 0.000). This implies that RQ2 is not confirmed. Here our third result:

***Result 3*** When the trustor cumulated earnings are higher than those of the trustee, this advantage has a significant negative effect on the trust decision, sending a lower amount to the trustee. No significant effect is found otherwise.

Additionally, to catch a possible treatment effect, we include dummy variables in our analysis, where Treatment B is taken as the reference treatment. Only Treatment E is found to be statistically significant. In other words, in Treatment E the trustor sends an amount (10% lower) that is significantly different (see Table 9, column 1) from that of the trustor in Treatment B. On the contrary, no statistical differences are found between trustors’ decisions in Treatments H and B. This is related to our second research question, and allows us to state that:

***Result 4*** Trust is found to be significantly lower in Treatment E than in Treatment B. No other differences between treatments are found with respect to the trust decisions.

Finally, our analysis on the influence of personality traits on the trustors’ decision finds statistically significant positive differences between altruist trustors and inequality loving ones.

### Trustees’ Reciprocity

We estimate random-effects ordered logistic regression with cluster–robust standard errors for panels nested within groups. For the analysis of the trustees’ decisions, we have created three dependent variables (levels L1, L2, L3), associated with the egalitarian strategy,^14^ that take values 1, 2, 3, respectively, indicating that the trustee returns an amount lower than (L1), equal to (L2) or higher than (L3) the egalitarian amount, respectively. Table 9 reports the coefficients and marginal effects.

Regarding the relationship between the amount sent by the trustor and the amount returned by the trustee, we find a negative and significant coefficient in the regression which implies that, in general terms, the higher the amount sent by the trustor, the lower the (total) amount sent back by the trustee. Taking as a reference the egalitarian amount and differentiating by levels, we find a positive and significant marginal effect at level L1; that is, an increase in the amount sent by the trustor makes more likely that the trustee returns an amount lower than the egalitarian (2.37%, *p* = 0.000). On the contrary, the marginal effects associated to the decision at levels L2 and L3 are significant but negative: an increase in the amount sent by the trustor makes less likely that the trustee returns the egalitarian (−1.56%, *p* = 0.000) or higher than the egalitarian (−0.81%, *p* = 0.001) amount. Therefore, it may be concluded that:

***Result 5*** The probability of reciprocating with a higher or equal (lower) than the egalitarian amount decreases (increases) with the trust rate.

With respect to Treatment E, it is important to highlight that when the endowment of the trustee is higher (lower) than that of the trustor, the marginal effect is positive and significant (0.53%, *p* = 0.000) (negative and significant: −0.87%, *p* = 0.000), increasing (decreasing) the probability of returning a lower than the egalitarian amount. Also significant but the opposite is observed at levels L2 and L3, where the estimated probability decreases (increases) by 0.34% (0.57%) and 0.18% (0.3%), respectively, when the trustee has an initial endowment higher (lower) than that of the trustor. In other words, the initial endowment inequality has an effect on L2 or L3 reciprocity decisions that is the opposite to the inequality sense. The opposite is found in L1. Summarizing:

***Result 6*** Reciprocity is affected by the endowment inequality in the TG. Specifically, the probability that the trustee reciprocates with an amount equal or higher than the egalitarian increases (decreases) when he is the one with the lower (higher) endowment.

Observe that our Result 6 contradicts the second part of RQ2. That is, the trustee’s decisions are affected not only by the amount received from the trustor but also by the endowment heterogeneity. Previous literature suggests that the trustee’s decisions in the TG are affected by his psychological characteristics (Attanasi and Nagel, 2008; Andrighetto et al., 2015; Di Bartolomeo and Papa, 2016).

Somehow the opposite occurs in Treatment H. Specifically, in the case in which the trustee’s cumulated earnings are higher (lower) than those of the trustor, the marginal effect is negative and significant (−0.01%, *p* = 0.052) (negative and significant: −2.34%, *p* = 0.000), decreasing the probability of returning amounts lower than the egalitarian. For levels L2 and L3 we observe the opposite: the estimated probability significantly increases, respectively, by 0.01% (*p* = 0.054) and 0.005% (*p* = 0.062) when the trustee’s cumulated earnings are higher than those of the trustor. A significant increase is also estimated when the trustee’s cumulated earnings are lower than those of the trustor in L2 (1.54%, *p* = 0.000) and L3 (0.8%, *p* = 0.000). Consequently, the cumulated earnings inequality exhibits a positive (negative) effect on the probability of taking an egalitarian or superior (inferior) reciprocity decision.

***Result 7*** Inequality in accumulated earnings affects the reciprocity decision in the TG. In particular, the probability of reciprocating with an amount lower than the egalitarian increases (decreases) only when the trustee’s accumulated earnings are higher (lower) than those of the trustor. The probability of reciprocating with an amount equal or higher than the egalitarian increases independently on who is richer/poorer.

Two trustees’ personality archetypes are found to be statistically significant with respect to the egalitarian strategy: the altruist and the inequality-adverse. Specifically, the altruist is more likely than any other archetype to reciprocate with the egalitarian. Surprisingly, the inequality-adverse is significantly less likely to do that.

Finally, we have estimated the probability of reciprocating for each reciprocity level: 77.1% (in L1) 17.3% (in L2) and 5.6% (in L3). It is not surprising that, given that our sample of trustees is highly represented by selfish and inequality-lovers, the more likely decision has been to reciprocate with an amount that is lower than the egalitarian.

### Trustees’ Altruism

We estimate random-effects binary logit regression with cluster–robust standard errors for panels nested within groups. The dependent variable (see Table 8) takes value 1 indicating that the trustee sends a positive amount from his own initial endowment, and value 0 otherwise. Observe Table 9 for the coefficients and marginal effects.

First, a general positive and significant relationship is found between trust and altruism decisions, with a positive and significant marginal effect (0.0054, *p* = 0.004), indicating a positive effect on the probability of being altruistic in our design. In fact, it is also observed that how much the trustee gives in the altruism decision does not depend from how much he reciprocates. Therefore:

***Result 8*** Independently on the treatment, the altruism decision of the trustee is positively related to the trust decision. Moreover, the altruism decision does not depend on the reciprocity decision, but exclusively on the trustor’s decision.

Second, in Treatment E the initial endowment inequality plays a significant positive role in the probability of sending a positive amount in the altruism decision only when the trustee’s endowment is higher than that of the trustor’s (0.0033, *p* = 0.000). For Treatment H the opposite result is found, that is, when the trustee’s cumulated earnings are higher than those of the trustor, there is a significant negative effect on the probability of the trustee adopting an altruistic decision (−0.0002, *p* = 0.007). As a result:

***Result 9*** In the altruism stage, it is more (less) likely that the trustee sends a positive amount when he has a higher initial endowment (cumulated earnings) than his corresponding trustor. No significant marginal effect is found otherwise.

Finally, our analysis on the influence of personality traits on the trustees’ decision on altruism finds that psychological variables related to inter-personal trust and reciprocity^15^ exhibit the highest marginal effects on the probability of sending money in the altruism decision. However, the EET archetypes show no statistical significance.

## Discussion and Main Conclusion

Our motivation for this paper has been to study the importance of economic heterogeneity in the decision of how much to trust and reciprocate. Our hypothesis is that individuals assign a different value to income resulting from their own effort than to income received without dedicating energy to it in the case of inheritance or a subsidy. In other words, endogenous economic heterogeneity plays a role in trust and reciprocity behavior. Moreover, we have put this endogenous source of inequality in contrast with an exogenous source of economic inequality, that is the case in which individuals have accumulated different amount of money over time. We also have hypothesized that being aware of such economic heterogeneity can affect the trust and/or reciprocity levels of individuals.

To the best of our knowledge, our work is the first in designing a situation in which trust, reciprocity and altruism are analyzed taking into consideration those sources of economic heterogeneity: endogenous through real-effort and exogenous as result of different accumulated earnings. With an experiment in which a finitely repeated version of the TG is at the core of the design, this paper has analyzed whether the levels of trust, reciprocity and altruism are affected by heterogeneity on accumulated earnings and/or initial endowment. Two treatments in our design allow for testing how the fact of knowing how rich the partner is or how well the partner performed several tasks with real effort may really affect the trust, reciprocity or altruism levels in a TG environment. On one hand, a Treatment H in which the cumulated earnings are common knowledge at the end of each round, has made the subjects aware of any income heterogeneity throughout the TG. On the other hand, an alternative Treatment E has introduced three initial real-effort tasks which generate endowment heterogeneity kept fixed at the beginning of each round in the TG. A clear treatment effect has been found confirming that the trust level is endowment as well as cumulated earnings dependent.

A baseline Treatment B with no endowment heterogeneity is taken as a reference. In the three treatments, the trustee takes two decisions: reciprocity and altruism decision. A general result that does not depend on the treatment is that trust decisions are aligned with recent past experience. More specifically, except for the first period, the amount sent to the trustee in one period positively depends on the amount returned from the corresponding trustee in the two previous periods. Interestingly, a positive experience received from the trustee has a positive effect in the attitude of the trustor toward the new trustee(s) in the next round(s). Such experience effect obtained under random matching protocol somehow extends previous results obtained by Attanasi et al. (2019) under partner matching.

In general terms, the literature agrees on the fact that wealth inequality reduces incentives to cooperate (Ciriolo, 2007; Heap et al., 2013; Gallego, 2016). Specifically, Bejarano et al. (2018) show that inequality reduces trust levels only when this inequality is generated by random shocks. Our results show that inequality significantly reduces trust in Treatment E. Also, from the trustee’s perspective, the endowment inequality generated in Treatment E affects negatively the levels of reciprocity that are higher or equal to the egalitarian strategy, the one that assures that trustor and trustee enjoy the same payoffs. Previous literature provides evidence of negative effects on reciprocity under different contexts. For instance, Pelligra et al. (2020) find that trustors’ expectations do not always have a positive effect on trustees’ decision, that is, trustors’ expectations expressed as request not always increase reciprocity. Furthermore, Balafoutas and Fornwagner (2017), using a Dictator Game, find that the guilt aversion only affects decisions up to a certain level of recipient expectations. As a result, RQ2 is confirmed.

The present work has addressed also the effect of the inequality direction -which player is the richest-on the levels of trust, reciprocity and altruism. Our results on this diverge from those of Ciriolo (2007); Brülhart and Usunier (2012), and Rodriguez-Lara (2018) who find a non-significantly different behavior when facing poorer/richer partners compared to the case of equality. More in line with Xiao and Bicchieri (2010) and Smith (2011b), our analysis results in significant effects of inequality direction on trust and reciprocity levels, especially in the treatment with effort. Surprisingly, whenever the trustor’s endowment is higher (lower) than the trustee’s, trust levels decrease (increase). It seems that having performed better than the partner in the tasks affects trust in a negative way, maybe because the trustor anticipates that the trustee will be less willing to send money back. Furthermore, the altruism decision does not depend on the reciprocity decision, but exclusively on the trustor’s decision.

From the perspective of the trustee, the contrary effect of inequality direction is found to be significant with respect to the altruism level in Treatments E and H. Specifically, when the initial endowment is higher than that of the trustor, the trustee behaves more altruistically, sending a higher part of his initial endowment. The contrary effect is found in Treatment H, therefore confirming that the effect of deserving the endowment with effort has a positive effect in the intention of being altruistic, thus decreasing the inequality between partners. Somehow comparable is the result of Engel (2011) in his metanalysis of the Dictator Game where he finds that when the dictator has to earn the pie, or the recipient has his own endowment, generosity significantly decreases. Although not in the altruism’ decision, we also find a negative effect of effort on the trust levels.

Interestingly, addressing the relationship between trust and reciprocity decisions with some personality archetypes, authors like Espín et al. (2016) and Bellucci et al. (2019) find considerable heterogeneity in the TG decisions as well as in social preferences or motivation. Regarding the four archetypes identified with the EET-pre, it is surprising that although altruist and inequality-lover trustors present positive significant differences, the altruists and the spiteful trustors do not. On the trustees’ type, the inequality-averse presents a negative marginal effect, decreasing the probability of taking the egalitarian strategy, contrary to expected. The altruistic archetype does show a positive marginal effect on the reciprocity decision, using more likely the egalitarian strategy.

In summary, the data analysis highlights the importance of past accumulated earnings levels (Treatment H) as well as endowment heterogeneity (Treatment E) on the actual levels of trust, reciprocity and altruism. Specifically, it is observed that the decision of trustors is positively affected by positive past experienced reciprocity. Moreover, trustors are sensitive to how much money the trustee accumulates each round, trusting more the ones that have less compared to themselves. The salient result in Treatment E is that trustors are sensitive to the endowment level of the trustees, trusting more the partners that have got a higher than own endowment, probably considering that a person that performed better in the tasks is a better partner to trust.

A gender analysis of our data, although without significant differences to remark, confirms a result previously found in the literature (Buchan et al., 2008; Dittrich, 2015): women trust in median less than males, although this gender effect vanishes when the endowment is the result of own effort in real tasks, where the significant gender difference is found in the role of the trustee, being females the ones that reciprocate less than males in Treatment E. One could say that the importance that women give to getting the endowment with own effort is stronger if they play the role of trustees rather than the trustor.

Of course, our results go in line with any policy measures that focus on minimizing economic inequality, since its importance goes beyond unexpected limits that affect social welfare.

## Data Availability Statement

The raw data supporting the conclusions of this article will be made available by the authors, without undue reservation.

## Ethics Statement

The studies involving human participants were refereed and approved by the Laboratorio de Economía Experimental, Universitat Jaume I, Castellón, Spain. The participants provided their written informed consent to participate in this study.

## Author Contributions

AR-G contributed to the experimental design, programmed the software of the experiment, and coordinated the data analysis and made a first draft of the manuscript. MC-T built the review of the literature and was also involved in the design and in the writing of the final version of the manuscript. AG-G coordinated the experimental design and run the sessions in the lab and was deeply involved in the writing of the final version of the manuscript. All authors were involved in the writing of the final version of the manuscript and approved the submitted version.

## Conflict of Interest

The authors declare that the research was conducted in the absence of any commercial or financial relationships that could be construed as a potential conflict of interest.

## Publisher’s Note

All claims expressed in this article are solely those of the authors and do not necessarily represent those of their affiliated organizations, or those of the publisher, the editors and the reviewers. Any product that may be evaluated in this article, or claim that may be made by its manufacturer, is not guaranteed or endorsed by the publisher.

## Footnotes

1 The trustee observes two boxes on the screen, both preceded by the corresponding question: (1) How much of the amount received do you want to return to the trustor? (2) How much from your endowment do you want to send to the trustor?

2 It was unlikely that the subject could answer the 50 questions before the time was over.

3 A positive (negative) *x-score* corresponds to benevolent (malevolent) behavior in the domain of disadvantageous inequality, whereas a positive (negative) *y-score* corresponds to benevolent (malevolent) behavior in the domain of advantageous inequality. Both scores vary from −2.5 to +2.5 (given that there are 5 questions in each test), being the relevant magnitude the number of times in which the subject chooses RIGHT(*x-score*)/LEFT(*y-score*). Therefore, the correspondence of each archetype and score interval is: altruist (x > 0, y > 0); inequality loving (x > 0, y < 0); spiteful (x < 0, y < 0); inequality adverse (x < 0, y > 0).

4 The questions are available from the authors upon request.

5 Evans and Revelle (2008) use “The propensity to trust survey (PTS)” and find evidence that trust and trustworthiness are compound personality traits, and that PTS scales are preferable to general Big Five measures for predicting trusting behavior.

6 Caliendo et al. (2012, 2014) use the German Socio-Economic Panel (SOEP).

7 Spreng et al. (2009) use the Toronto Empathy Questionnaire (TEQ).

8 Other authors like Attanasi et al. (2019) underline the relevance of the reputation effect on players’ decisions. According to them, reputation is a significant motivation that positively affects share decisions under a partner matching context, unlike our random matching design where the reputation effect is blurred.

9 Specifically, the weights were chosen considering that task 1 is the easiest and task 2 is the more difficult for an average person. Even though not directly, the criteria we followed are related with the concepts of control and routine processing indicated in Goldhammer et al. (2014). Task 1 is considered the easiest, since it just requires visual speed; task 3 requires a more routine processing; task 2 includes more difficulty, since it demands for more control processing.

10 The TG-effect on EET is measured through a contrast in the global sample (exact symmetry test, *p* = 0.0198; Stuart-Maxwell (SM) test for marginal homogeneity, chi2 = 7.57, df = 3, *p* = 0.0558). Such an effect exists in Treatment B (exact symmetry test, *p* = 0.0430; SM-test for marginal homogeneity, chi2 = 8.17, df = 3, *p* = 0.0426), and in Treatment H (exact symmetry test, *p* = 0.0608; SM-test, chi2 = 8.47, df = 3, *p* = 0.0372). No TG-effect on EET is found in TE (exact symmetry test, *p* = 0.3579; SM-test, chi2 = 2.06, df = 3, *p* = 0.5592).

11 See also Table A in the Supplementary Material file for details on trustors’ decisions by gender. Furthermore, no significant gender differences are found in Treatment E.

12 An ex-post power analysis has been conducted using Stata with power set at 0.80 and probability at 0.05. It is obtained that the sample size necessary for statistically significant differences at 5% between baseline and treatment should be at least *N* = 239 observations.

13 Results from the EET-post are not included in this analysis, given that the number of inconsistencies in the post experiment test was enough to un-equilibrate our number of observations and, therefore, the interest in the comparability. For the purpose of the econometric analysis, we believe that the spirit of the test is more genuinely captured by the decisions taken in the EET-pre.

14 A trustee that follows this strategy chooses the amount y1 such that the payoffs of both players are equal that round. This implies that trustor’s payoff *π_*or*_ = E-x+y_1_* has to be equal to the trustee’s *π_*ee*_ = 3x*–*y_1_ + E*. In TB, the egalitarian amount is: *y_1_ = 2x*. In Treatment H, the egalitarian amount is balanced by the earnings inequality: *y_1_ = 2x + (π_*ee*_*–*π_*or*_)/2*. In Treatment E, the egalitarian amount considers the endowment inequality, i.e., equal payoffs imply that *E*_*or*_–*x+y_1_ = 3x*–*y_1_ + E_ee_*; therefore, the egalitarian amount in Treatment E is *y_1_ = 2x + (E_ee_*–*E_or_)/2*. In all cases, the amount sent by the trustee has to fulfill the non-transfer restriction along rounds and, therefore, *y*_1_ € [0, 3x].

15 Negative-reciprocity and negative-trust (see Table 9).

## References

[B1] Abeler J. Falk A. Goette L. Huffman D. (2011). Reference points and effort provision. *Am. Econ. Rev.* 101 470–492. 10.1257/aer.101.2.470

[B2] Alesina A. La Ferrara E. (2002). Who trusts others?. *J. Public Econ.* 85 207–234. 10.1016/S0047-2727(01)00084-6

[B3] Anderson L. R. Mellor J. M. Milyo J. (2006). Induced heterogeneity in trust experiments. *Exp. Econ.* 9 223–235. 10.1007/s10683-006-9124-2

[B4] Andreoni J. Nikiforakis N. Stoop J. (2017). *Are the Rich More Selfish Than The Poor, Or Do They Just Have More Money? A Natural Field Experiment.* Cambridge: National Bureau of Economic Research. 10.3386/w23229

[B5] Andrighetto G. Grieco D. Tummolini L. (2015). Perceived legitimacy of normative expectations motivates compliance with social norms when nobody is watching. *Front. Psychol.* 6:1413. 10.3389/fpsyg.2015.01413 26500568 PMC4593938

[B6] Attanasi G. Battigalli G. Manzoni E. Nagel R. (2019). Belief-dependent preferences and reputation: experimental analysis of a repeated trust game. *J. Econ. Behav. Organ.* 167 341–360.

[B7] Attanasi G. Battigalli G. Nagel R. (2013). *Disclosure of Belief-Dependent Preferences in the Trust Game.* Milano: Bocconi University.

[B8] Attanasi G. Nagel R. (2008). “A survey of psychological games: theoretical findings and experimental evidence,” in *Games, Rationality and Behaviour, Essays in Behavioural Game Theory and Experiments*, Eds Innocenti A. Sbriglia P., (London: Palgrave Macmillan), 204–232.

[B9] Balafoutas L. Fornwagner H. (2017). The limits of guilt. *J. Econ. Sci. Assoc*. 3 137–148.

[B10] Bejarano H. Gillet J. Rodriguez-Lara I. (2021a). Trust and trustworthiness after negative random shocks. *J. Econ. Psychol.* 86:102422

[B11] Bejarano H. Gillet J. Rodriguez-Lara I. (2021b). When the rich do (not) trust the (newly) rich: experimental evidence on the effects of positive random shocks in the trust game. *OSF* [Preprint]. 10.31219/osf.io/wmejt

[B12] Bejarano H. Gillet J. Rodriguez-Lara I. (2018). Do negative random shocks affect trust and trustworthiness? *South. Econ. J.* 85 563–579. 10.1002/soej.12302

[B13] Bellucci G. Hahn T. Deshpande G. Krueger F. (2019). Functional connectivity of specific resting-state networks predicts trust and reciprocity in the trust game. *Cogn. Affect. Behav. Neurosci.* 19 165–176. 10.3758/s13415-018-00654-3 30357662

[B14] Berg J. Dickhaut J. McCabe K. (1995). Trust, reciprocity, and social history. *Games Econ. Behav.* 10 122–142. 10.1006/game.1995.1027

[B15] Blanco M. Dalton P. (2019). *Generosity and Wealth: Experimental Evidence from Bogotá Stratification.* Bogotá: Universidad del Rosario.

[B16] Bornhorst F. Ichino A. Kirchkamp O. Schlag K. H. Winter E. (2010). Similarities and differences when building trust: the role of cultures. *Exp. Econ.* 13 260–283.

[B17] Brülhart M. Usunier J. C. (2012). Does the trust game measure trust? *Econ. Lett.* 115 20–23. 10.1016/j.econlet.2011.11.039

[B18] Buchan N. R. Croson R. T. Solnick S. (2008). Trust and gender: an examination of behavior and beliefs in the Investment Game. *J. Econ. Behav. Organ.* 68 466–476. 10.1016/j.jebo.2007.10.006

[B19] Caliendo M. Fossen F. Kritikos A. S. (2014). Personality characteristics and the decisions to become and stay self-employed. *Small Bus. Econ.* 42 787–814.

[B20] Caliendo M. Fossen F. M. Kritikos A. S. (2012). Trust, positive reciprocity, and negative reciprocity: do these traits impact entrepreneurial dynamics? *J. Econ. Psychol.* 33 394–409. 10.1016/j.joep.2011.01.005

[B21] Chetty R. Hofmeyr A. Kincaid H. Monroe B. (2020). The Trust Game does not (only) measure trust: the risk-trust confound revisited. *J. Behav. Exp. Econ.* 90:101520. 10.1016/j.socec.2020.101520

[B22] Ciriolo E. (2007). Inequity aversion and trustees’ reciprocity in the trust game. *Eur. J. Polit. Econ.* 23 1007–1024. 10.1016/j.ejpoleco.2006.01.001

[B23] Coane J. H. Umanath S. (2021). A database of general knowledge question performance in older adults. *Behav. Res. Methods* 53 415–429. 10.3758/s13428-020-01493-2 33443730 PMC7880974

[B24] Corgnet B. Espín A. M. Hernán-González R. Kujal P. Rassenti S. (2016). To trust, or not to trust: cognitive reflection in trust games. *J. Behav. Exp. Econ.* 64 20–27. 10.1016/j.socec.2015.09.008

[B25] Cox J. C. (2004). How to identify trust and reciprocity. *Games Econ. Behav.* 46 260–281. 10.1016/S0899-8256(03)00119-2

[B26] Cox J. C. Kerschbamer R. Neururer D. (2016). What is trustworthiness and what drives it? *Games Econ. Behav.* 98 197–218. 10.1016/j.geb.2016.05.008

[B27] Di Bartolomeo G. Papa S. (2016). Trust and reciprocity: extensions and robustness of triadic design. *Exp. Econ.* 19 100–115.

[B28] Dittrich M. (2015). Gender differences in trust and reciprocity: evidence from a large-scale experiment with heterogeneous subjects. *Appl. Econ.* 4736 3825–3838. 10.1080/00036846.2015.1019036

[B29] Engel C. (2011). Dictator games: a meta study. *Exp. Econ*. 14 583–610.

[B30] Espín A. M. Exadaktylos F. Neyse L. (2016). Heterogeneous motives in the trust game: a tale of two roles. *Front. Psychol.* 7:728. 10.3389/fpsyg.2016.00728 27242633 PMC4870259

[B31] Evans A. M. Revelle W. (2008). Survey and behavioral measurements of interpersonal trust. *J. Res. Pers.* 42 1585–1593.

[B32] Fehr D. (2018). Is increasing inequality harmful? Experimental evidence. *Games Econ Behav.* 107 123–134. 10.1016/j.geb.2017.11.001

[B33] Fehr D. Rau H. Trautmann S. T. Xu Y. (2020). Inequality, fairness and social capital. *Eur. Econ. Rev.* 129:103566. 10.1016/j.euroecorev.2020.103566

[B34] Fischbacher U. (2007). z-Tree: zurich toolbox for ready-made economic experiments. *Exp. Econ.* 10 171–178. 10.1007/s10683-006-9159-4

[B35] Gallego A. (2016). Inequality and the erosion of trust among the poor: experimental evidence. *Soc. Econ. Rev.* 14 443–460. 10.1093/ser/mww010

[B36] Goldhammer F. Naumann J. Stelter A. Tóth K. Rölke H. Klieme E. (2014). The time on task effect in reading and problem solving is moderated by task difficulty and skill: insights from a computer-based large-scale assessment. *J. Educ. Psychol.* 106 608–626. 10.1037/a0034716

[B37] Greiner B. (2015). Subject pool recruitment procedures: organizing experiments with ORSEE. *J. Econ. Sci. Assoc.* 1 114–125. 10.1007/s40881-015-0004-4

[B38] Greiner B. Ockenfels A. Werner P. (2012). The dynamic interplay of inequality and trust—an experimental study. *J. Econ. Behav. Organ.* 81 355–365. 10.1016/j.jebo.2011.11.004

[B39] Heap S. P. H. Tan J. H. Zizzo D. J. (2013). Trust, inequality and the market. *Theory Decis.* 74 311–333. 10.1007/s11238-011-9287-y

[B40] Holzmeister F. Kerschbamer R. (2019). oTree: the equality equivalence test. *J. Behav. Exp. Finance* 22 214–222. 10.1016/j.jbef.2019.04.001

[B41] Johnson N. D. Mislin A. A. (2011). Trust games: a meta-analysis. *J. Econ. Psychol.* 32 865–889. 10.1016/j.joep.2011.05.007

[B42] Kerschbamer R. (2015). The geometry of distributional preferences and a non-parametric identification approach: the equality equivalence test. *Eur. Econ. Rev.* 76 85–103. 10.1016/j.euroecorev.2015.01.008 26089571 PMC4459445

[B43] Khalmetski K. Ockenfels A. Werner P. (2015). Surprising gifts: theory and laboratory evidence. *J. Econ. Theory* 159 163–208.

[B44] Lei V. Vesely F. (2010). In-group versus out-group trust: the impact of income inequality. *South. Econ. J.* 76 1049–1063.

[B45] Mohnen A. Pokorny K. Sliwka D. (2008). Transparency, inequity aversion, and the dynamics of peer pressure in teams: theory and evidence. *J. Labor Econ.* 26 693–720.

[B46] Niederle M. Vesterlund L. (2007). Do women shy away from competition? Do men compete too much? *Q. J. Econ.* 122 1067–1101. 10.1162/qjec.122.3.1067

[B47] Pelligra V. Reggiani T. Zizzo D. J. (2020). Responding to (un)reasonable requests by an authority. *Theory Decis.* 89 287–311.

[B48] Putnam R. (2000). *Bowling Alone: The Collapse And Revival Of American Community.* New York: Simon and Schuster paperbacks.

[B49] Rodriguez-Lara I. (2018). No evidence of inequality aversion in the investment game. *PLoS One* 13:e0204392. 10.1371/journal.pone.0204392 30352052 PMC6198942

[B50] Smith A. (2011a). Identifying in-group and out-group effects in the trust game. *BE J. Econ. Anal. Policy* 11 1–13. 10.2202/1935-1682.2878

[B51] Smith A. (2011b). Income inequality in the trust game. *Econ. Lett.* 111 54–56. 10.1016/j.econlet.2011.01.008

[B52] Spreng R. N. McKinnon M. C. Mar R. A. Levine B. (2009). The Toronto Empathy Questionnaire: scale development and initial validation of a factor-analytic solution to multiple empathy measures. *J. Person. Assess.* 91 62–71. 10.1080/00223890802484381 19085285 PMC2775495

[B53] Xiao E. Bicchieri C. (2010). When equality trumps reciprocity. *J. Econ. Psychol.* 31 456–470. 10.1016/j.joep.2010.02.001

[B54] Yan J. Miao L. (2007). “Effects of endowments on reciprocal behaviors,” in *2007 International Conference on Wireless Communications, Networking and Mobile Computing*, (Manhattan: IEEE), 4591–4594.

