# Supplementary material for: Effects of Inequality on Trust and Reciprocity: An Experiment With Real Effort
Source: Front Psychol. 2021 Dec 2;12:745948. doi: 10.3389/fpsyg.2021.745948 (PMC8674586; doi:10.3389/fpsyg.2021.745948)
Supplement: Supplementary file 1 [file Data_Sheet_1.docx]

Supplementary Material

# Experimental instructions

# This section includes the instructions given to experimental subjects, translated from the original in Spanish. The experimental session consisted of two parts. Part I was dedicated to the tasks that the subjects had to perform in the first place, and the instructions of this part were displayed on each computer’s screen, in a sequential way task by task. After that, Part II started with subjects receiving a hard copy of the instructions related with the TG which were also read aloud by the experimenter.

# In the following we include the contain the text of the general instructions and then the written version of the baseline treatment (BT) of Part II, including in parenthesis the details that are specific to the other two treatments, TH and TE.

# TB – Baseline treatment of the TG (details of TH within brackets)

You are going to participate in an experimental session that will give you money in cash. The amount that you can get will depend on your decisions and the decisions of the other participants in this room. Please switch off your mobile and put your things away. You will need just the instructions and the computer. Please rise your hand in case you have questions and the experimenter will attend you privately.

In this part there are 2 players, 1 and 2, and you will be one of them. Your type will be randomly assigned by the computer and will remain fixed during the whole session.

Both players have an initial endowment of 50 ExCUs (experimental money) and this is common knowledge.

*Decision making*

- In a first stage, **player 1** has to decide on how much of her endowment to send to player 2, knowing that any amount she gives will be multiplied by 3 in player 2’s hands.

For example, if player 1 decides to send Y ExCUs to player 2, player 2 will receive the amount of 3Y ExCUs. Therefore, player 2 will keep 50+3Y ExCUs in his hands.

- In a second stage, after observing her decision, player 2 has to decide how much to send to player 1. Specifically, **player 2**’s decisions are two:

**Decision 1**. How much does he want to send to player 1 from the total received (3Y ExCUs in our example)?

**Decision 2**. How much does he want to send to player 1 from his initial endowment (50 ExCUs)?

Using the same example, if player 2 decides to send nothing to player 1, player 2 will keep a total of 50+3Y ExCUs and player 1 a total of 50-Y ExCUs.

Each round, after both players decide, player 1 will be informed about the amount sent by player 2 and both players will receive information about own money earned in that round and own money accumulated so far but no information will be given about the money of the partner. *[Only TH:…both players will receive information about the money earned that round as well as the money accumulated so far (own and the partner’s)]*

This same situation will be repeated during 12 rounds. Your type will be the same during the whole session, but the matching will be random in each period, that is, each round you will play with a different player.

Finally, a period will be randomly selected by the computer to calculate your earnings in this part. The amount in euro you will get will be equal to 10% of the money you earned in that period.

After round 12, your screen will show a questionnaire. A fixed amount of euro will be paid to you for answering the questions. At the end of the session, you will see the total earnings on your screen and you will be paid in cash privately on the lab’s counter.

# TE – Treatment with Effort

You are going to participate in an experimental session that has two parts and that will give you the possibility to earn some money in cash. The amount that you can get will depend on your decisions and the decisions of the other participants in the room corresponding to the two parts. Please switch off your mobile and put your things away. You will need just the instructions and the computer. Please rise your hand in case you have questions and the experimenter will attend you privately.

**PART I**

The first part consists of three differentiated tasks. At the beginning of each task you will see on your screen the specific instructions about what it is and what do you have to do. After performing the three tasks, you will see on the screen the result you obtained in each task. That result will determine your initial endowment for you decision making in part II.

[On the screen of each computer, the following appears in this part:

# PART I. Instructions

This part of the experiment consists of three task that are described in the following:

**TASK 1.** In this task, matrices with zeros and ones are going to appear on your computer screen during 3 minutes. In a sequential way, you will see a 6x6 matrix on your screen and your task is to count and write the number of ones of the matrix. Independently on whether or not you answer is correct, you will tick “NEXT” and carry on with the next task.

**TASK 2.** In this task, two-digit numbers will appear on your screen during 3 minutes. In a sequential way, you will see 4 numbers of two digits each that you will have to add up. Independently on whether or not you answer is correct, you will tick “NEXT” and carry on with the next task.

**TASK 3.** In this task, several questions of general knowledge will appear on your screen during 2.5 minutes. In a sequential way you will see a multiple-choice question with four options. Please tick what you think is the correct option. Independently on whether or not you answer is correct, you will tick “NEXT” and carry on with the next task.]

**PART II**

In this part there are 2 players, 1 and 2, and you will be one of them. Your type will be randomly assigned by the computer and will remain fixed during the whole session.

Each player will have an initial endowment that depends on her performance in the tasks of Part I, denoted as: X_1_ for player 1 and X_2_ for player 2. This endowment, expressed in ExCUs (experimental money), will be the same at the beginning of each round. The endowment is common knowledge for players.

*Decision making*

Each round, decision making consist in:

- First, **player 1** has to decide on how much of her endowment to send to player 2, knowing that any amount she gives will be multiplied by 3 in player 2’s hands.

For example, if player 1 decides to send Y ExCUs to player 2, player 2 will receive the amount of 3Y ExCUs. Therefore, player 2 will keep X_2_+3Y ExCUs in his hands.

- Second, after observing her decision, player 2 has to decide how much to send to player 1. Specifically, **player 2**’s decisions are two:

**Decision 1**. How much does he want to send to player 1 from the total received (3Y ExCUs in our example)?

**Decision 2**. How much does he want to send to player 1 from his initial endowment (X_2_ ExCUs)?

Using the same example, if player 2 decides to send nothing to player 1, player 2 will keep a total of X_2_+3Y ExCUs and player 1 a total of X_1_-Y ExCUs.

Each round, after both players decide, player 1 will be informed about the amount sent by player 2 and both players will receive information about own money earned in that round and own money accumulated so far but no information will be given about the money of the partner.

This same situation will be repeated during 12 rounds. Your type will be the same during the whole session, but the matching will be random in each period, that is, each round you will play with a different player.

Finally, a period will be randomly selected by the computer to calculate your earnings in this part. The amount in euro you will get will be equal to 10% of the money you earned in that period.

After round 12, your screen will show a questionnaire. A fixed amount of euro will be paid to you for answering the questions. At the end of the session, you will see the total earnings on your screen and you will be paid in cash privately on the lab’s counter.

*Earnings*

At the end of the session, the amount in euro that you will receive will have three components:

From Part I you will receive an amount that will depend on the initial endowment you got.

From Part II you will be paid the 10% of the earnings you got in the random round chosen by the computer.

At the beginning as well as at the end of part II you will have to answer to a brief questionnaire. A fixed amount will be paid to you for answering these questions.

All these parts will conform the total earnings in euro that will be shown in your computer’s screen and that will be paid to you in cash privately on the counter.

# Supplementary Tables

The four next tables (A to D) show the main descriptive statistics of the decision of each player -trustor and trustee- in the TG for each treatment, differentiating also by gender. The decision of each player is expressed as the percentage sent to the partner. The notation used follows the one used in the TG of subsection 3.1 in the main manuscript. In particular, x represents the amount sent by the trustor; E is the initial endowment; G**m** denotes the own (m stands for **m**yself) cumulated earnings; Go denotes the other’s cumulated earnings; Em is the own (m stands for **m**yself) initial endowment. Eo is the other’s initial endowment.

**Table A.** Descriptive statistics of the *trustor*’s decision, by gender and treatment

| Stats. | **Trustor decision**  x/E =% of the initial endowment | | | | | | |
| --- | --- | --- | --- | --- | --- | --- | --- |
|  | **TB** | **TH** | | | **TE** | | |
|  | Total | Total | G_m_ ≤ G_o_ | G_m_ > G_o_ | Total | E_m_ ≤ E_o_ | E_m_ > E_o_ |
| Mean | 0.36 | 0.45 | 0.43 | 0.79 | 0.25 | 0.28 | 0.21 |
| Median | 0.2 | 0.4 | 0.40 | 1 | 0.13 | 0.15 | 0.10 |
| SD. | 0.35 | 0.36 | 0.35 | 0.28 | 0.29 | 0.30 | 0.26 |
| Obs. | 480 | 480 | 454 | 26 | 480 | 255 | 225 |
| **MALES** |  |  |  |  |  |  |  |
| Mean | 0.49 | 0.50 | 0.49 | 0.675 | 0.29 | 0.34 | 0.24 |
| Median | 0.42 | 0.40 | 0.40 | 0.75 | 0.08 | 0.11 | 0.06 |
| SD. | 0.38 | 0.39 | 0.39 | 0.35 | 0.35 | 0.37 | 0.33 |
| Obs. | 180 | 252 | 240 | 12 | 222 | 105 | 117 |
| **FEMALES** |  | . |  |  |  |  |  |
| Mean | 0.28 | 0.39 | 0.36 | 0.89 | 0.22 | 0.23 | 0.18 |
| Median | 0.15 | 0.32 | 0.30 | 1 | 0.15 | 0.15 | 0.14 |
| SD. | 0.30 | 0.31 | 0.29 | 0.19 | 0.21 | 0.30 | 0.18 |
| Obs. | 300 | 228 | 214 | 14 | 258 | 150 | 108 |

**Table B.** Descriptive statistics of the *trustee’s return rate*, by gender and treatment

| Stats. | **Trustee decision**  (y1 + y2)/x = total return rate | | | | | | |
| --- | --- | --- | --- | --- | --- | --- | --- |
|  | **TB** | **TH** | | | **TE** | | |
|  | Total | Total | G_m_ ≤ G_o_ | G_m_ > G_o_ | Total | E_m_ ≤ E_o_ | E_m_ > E_o_ |
| Mean | 1.19 | 1.32 | 1.42 | 1.30 | 1.27 | 1.26 | 1.27 |
| Median | 1 | 1.25 | 1.5 | 1.13 | 1 | 1 | 1.30 |
| SD. | 1.29 | 1.29 | .72 | 1.38 | 1.54 | 1.86 | 1.17 |
| Obs. | 423 | 407 | 66 | 341 | 445 | 214 | 231 |
| **MALES** |  |  |  |  |  |  |  |
| Mean | 1.19 | 1.26 | 1.29 | 1.25 | 1.43 | 1.54 | 1.37 |
| Median | 1 | 1.2 | 1.4 | 1.15 | 1.25 | 1 | 1.33 |
| SD. | 1.35 | 1.26 | .71 | 1.25 | 1.87 | 2.58 | 1.31 |
| Obs. | 288 | 257 | 39 | 218 | 259 | 94 | 165 |
| **FEMALES** |  |  |  |  |  |  |  |
| Mean | 1.18 | 1.43 | 1.61 | 1.40 | 1.05 | 1.04 | 1.05 |
| Median | 1 | 1.29 | 1.7 | 1.13 | 1 | 1 | 1 |
| SD. | 1.14 | 1.33 | 0.71 | 1.43 | 0.86 | 1.86 | 0.69 |
| Obs. | 135 | 150 | 27 | 123 | 186 | 120 | 66 |

**Table C.** Descriptive statistics of the trustee’s *reciprocity decision*, by gender and treatment

| Stats. | **Reciprocity decision**  y1/x = % returned to the trustor from the amount received | | | | | | |
| --- | --- | --- | --- | --- | --- | --- | --- |
|  | **TB** | **TH** | | | **TE** | | |
|  | Total | Total | G_m_ ≤ G_o_ | G_m_ > G_o_ | Total | E_m_ ≤ E_o_ | E_m_ > E_o_ |
| Mean | 0.75 | 0.93 | 10.09 | 0.89 | 0.81 | 0.74 | 0.88 |
| Median | 0.8 | 1 | 10.07 | 1 | 0.86 | 0.65 | 1 |
| SD. | 0.6 | 0.70 | 0.59 | 0.71 | 0.69 | 0.71 | 0.66 |
| Obs. | 423 | 407 | 66 | 341 | 445 | 214 | 231 |
| **MALES** |  |  |  |  |  |  |  |
| Mean | 0.76 | 0.92 | 10.02 | 0.90 | 0.85 | 0.73 | 0.92 |
| Median | 1 | 1 | 1 | 1 | 1 | 0.55 | 1 |
| SD. | 0.60 | 0.73 | 0.62 | 0.74 | 0.72 | 0.76 | 0.70 |
| Obs. | 288 | 257 | 39 | 218 | 259 | 94 | 165 |
| **FEMALES** |  |  |  |  |  |  |  |
| Mean | 0.74 | 0.95 | 10.2 | 0.89 | 0.75 | 0.75 | 0.78 |
| Median | 0.60 | 1 | 10.2 | 1 | 0.8 | 0.76 | 1 |
| SD. | 0.57 | 0.63 | 0.54 | 0.64 | 0.63 | 0.67 | 0.54 |
| Obs. | 135 | 150 | 27 | 123 | 186 | 120 | 66 |

**Table D.** Descriptive statistics of the trustee’s *altruism decision*, by gender and treatment

| Stats. | **Altruism decision**  y2/E = % sent to the trustor from the initial endowment | | | | | | |
| --- | --- | --- | --- | --- | --- | --- | --- |
|  | **TB** | **TH** | | | **TE** | | |
|  | Total | Total | G_m_ ≤ G_o_ | G_m_ > G_o_ | Total | E_m_ ≤ E_o_ | E_m_ > E_o_ |
| Mean | 0.12 | 0.14 | 0.19 | 0.13 | 0.08 | 0.11 | 0.061 |
| Median | 0.02 | 0 | 0.1 | 0 | 0 | 0 | 0 |
| SD. | 0.22 | 0.23 | 0.24 | 0.22 | 0.19 | 0.23 | 0.14 |
| Obs. | 480 | 480 | 69 | 411 | 480 | 230 | 250 |
| **MALES** |  |  |  |  |  |  |  |
| Mean | 0.14 | 0.11 | 0.13 | 0.11 | 0.08 | 0.11 | 0.06 |
| Median | 0.02 | 0 | 0.1 | 0 | 0 | 0 | 0 |
| SD. | 0.24 | 0.19 | 0.17 | 0.19 | 0.18 | 0.22 | 0.15 |
| Obs. | 324 | 312 | 41 | 271 | 280 | 104 | 176 |
| **FEMALES** |  |  |  |  |  |  |  |
| Mean | 0.08 | 0.20 | 0.28 | 0.18 | 0.09 | 0.11 | 0.05 |
| Median | 0.02 | 0.08 | 0.2 | 0.06 | 0 | 0.02 | 0 |
| SD. | 0.13 | 0.27 | 0.29 | 0.27 | 0.19 | 0.23 | 0.11 |
| Obs. | 156 | 168 | 28 | 140 | 200 | 126 | 75 |
